# Poliosis predicts durable response to immune-checkpoint blockade in metastatic melanoma: a multicenter cohort study

**DOI:** 10.1136/jitc-2026-015306

**Published:** 2026-10-05

**Authors:** Annika Brekner, Miriam Mengoni, Julian Kött, Berenice Mareen Lang, Alisa Lepper, Jakob Veeser, Janina Dietzel, Nora Laubach, Andrea Forschner, Christoffer Gebhardt, Stephan Grabbe, Henner Stege, Maximilian Haist

**Affiliations:** 1 Department of Dermatology, University Medical Center of the Johannes Gutenberg University Mainz, Mainz, Germany; 2 Department of Dermatology, Universitatsklinikum Schleswig-Holstein Campus Lubeck, Lübeck, Germany; 3 Department of Dermatology, Universitätsklinikum Magdeburg, Magdeburg, Germany; 4 Dermatology and Venereology, University Medical Center Hamburg-Eppendorf, Hamburg, Germany; 5 Fleur Hiege Center of Skin Cancer Research, University Medical Center Hamburg-Eppendorf, Hamburg, Germany; 6 Department of Dermatology, University Medical Center of the Goethe University Frankfurt, Frankfurt, Germany; 7 Division of Dermal Oncoimmunology, Helmholtz-Institute for Translational Oncology (HI-TRON), Mainz, Germany; 8 Department of Dermatology, University Hospital Tübingen, Tubingen, Germany; 9 Department of Microbiology and Immunology, Stanford University School of Medicine, Stanford, California, USA

**Keywords:** Melanoma, Immune Checkpoint Inhibitor, Immune related adverse event - irAE, Biomarker

## Abstract

**Background:**

Immune checkpoint blockade (ICB) improved survival outcomes for patients with metastatic melanoma. Yet up to 65% of patients experience primary resistance and nearly half of initial responders acquire resistance. The clinical utility of ICB is further tempered by frequent immune-related adverse events (irAEs), which increase in severity and frequency with prolonged treatment duration. Hence, a pivotal challenge is to identify clinically accessible markers to predict durable ICB responses. While prior reports indicate that the development of cutaneous irAEs may reflect robust antitumor immune responses, there is limited evidence linking specific types of cutaneous irAEs to clinical ICB benefit.

**Methods:**

In this retrospective multicenter study, we investigated a cohort of 741 patients diagnosed with non-resectable stage III/IV melanoma who were treated with ICB between April 2012 and August 2025. Patient demographics, tumor characteristics, treatment regimens and survival outcomes were collected within six German skin cancer centers. We stratified patients by the type of cutaneous AE into such with whitening of the body skin (vitiligo), hair (poliosis) or other cutaneous AE. Primary endpoints included progression-free survival (PFS) and objective response rates (ORR).

**Results:**

Among 741 eligible patients, 221 developed cutaneous irAE within the median follow-up period of 48 months. Thereof, we observed tumor recurrences in 17.2% of patients with poliosis, 57.7% of patients developing vitiligo and 60.0% of patients with other cutaneous irAE. In line, we detected a significantly enhanced ORR (93.1% vs 58.8% vs 38.8%, p*<*0.001), prolonged PFS (Not reached, NR, vs 31 months vs 9 months, p*<*0.001) and overall survival (NR vs NR vs 69 months, p<0.001) in poliosis patients. Critically, enhanced tumor control in poliosis patients was seen regardless of initial tumor burden, treatment duration or the presence of melanoma brain metastases. While other AE usually arise early on ICB start, poliosis was a late event that occurred at a median of 6 months.

**Conclusions:**

Our study identifies poliosis as a distinct and immunologically-informed marker linked to strong and durable ICB responses easily applicable in a routine clinical setting. Detection of poliosis may complement radiographic imaging for clinical decision-making on ICB treatment duration and early cessation and provides a window for translational investigations harnessing epitope-specific T-cell responses to melanoma antigens.

**WHAT IS ALREADY KNOWN ON THIS TOPIC**

- Despite the clinical success of immunotherapy in metastatic melanoma, at least two-thirds of patients will eventually develop a tumor relapse in most real-world patient cohorts. There is little data available on clinically accessible and cost-efficient biomarkers reliably predicting strong and durable immune checkpoint blockade (ICB) responses, thereby informing decision on treatment cessation.

**WHAT THIS STUDY ADDS**

- In this study, we show in a large, multicenter real-world patient cohort with non-resectable stage III/IV melanoma that the onset of poliosis during first-line ICB is associated with a significantly improved objective response rate and durable tumor remissions compared with vitiligo or other cutaneous adverse events.
- Critically, the onset of poliosis was linked to ICB benefit regardless of initial tumor burden or the presence of melanoma brain metastases.
- The development of poliosis is a slowly evolving cutaneous adverse event occurring after a median of 6 months independent from total ICB treatment duration.

**HOW THIS STUDY MIGHT AFFECT RESEARCH, PRACTICE OR POLICY**

- Our data suggest that the development of poliosis might serve as a distinct and immunologically informed biomarker linked to durable ICB responses that is easily applicable in a routine clinical setting to inform further treatment management strategies.

For the graphical abstract of this paper see figure 1.

**Figure 1:**
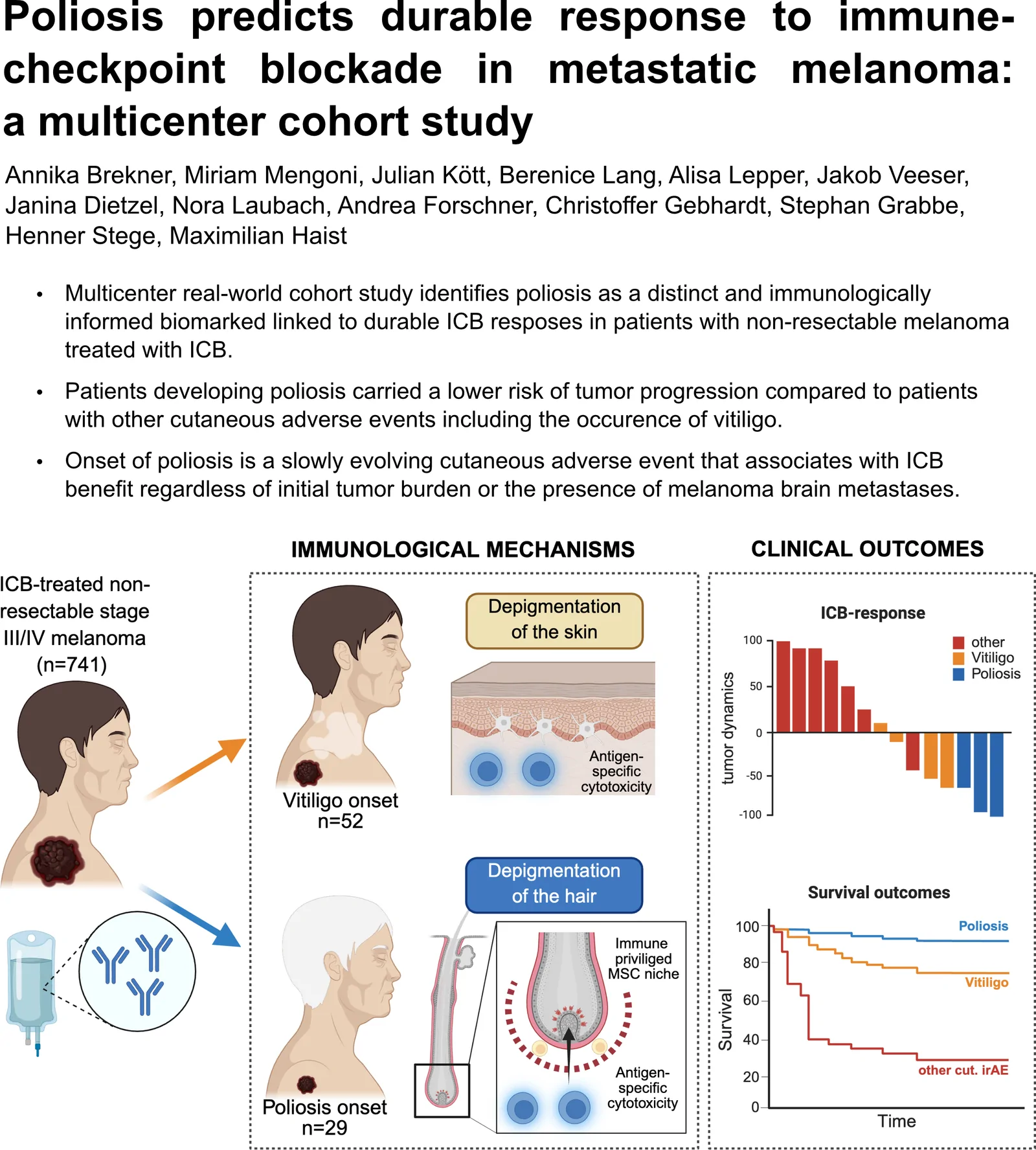
Graphical abstract.

## Background

 Immune checkpoint blockade (ICB) transformed the treatment landscape for patients with metastatic melanoma, contributing to markedly improved survival outcomes.^1 2^ Yet, 40–65% of patients do not derive benefit from ICB regimens due to primary ICB resistance and 45% of initial ICB responders will acquire resistance over time.^3^ Meanwhile, T-cell disinhibition evoked through ICB carries the risk of a number of immune-related adverse events (irAEs). In the CheckMate-067 study, which led to the approval of the combined therapy with ipilimumab and nivolumab, almost all patients (96%) treated with ipilimumab plus nivolumab experienced treatment-related AE, of which 59% were classified as severe (grade 3-4) according to Common Terminology Criteria for Adverse Events (CTCAE)^4^. Notably, the duration of ICB regimens is linked to the risk of developing severe and late-onset irAEs,^5 6^ which have significant implications for patients’ quality of life, healthcare costs, and long-term outcomes. While current Food and Drug Administration approvals specify treatment “until disease progression, unacceptable toxicity, or up to 24 months”, these recommendations for treatment duration lack robust evidence-based justification.

Therefore, a pivotal challenge is to identify clinically accessible markers that reliably predict strong and durable ICB responses that could be used for personalized decisions on the timing of treatment cessation.

Based on the assumption that the occurrence of irAE provides evidence for immunological activation through ICB administration, prior works reported that patients developing irAEs have higher response rates than patients without such events.^7–9^ However, these findings have not been universally verified and the current consensus is that irAE in general are not required to obtain ICB benefit.^10^

Recent works indicate that certain irAEs might be more directly related to antitumor efficacy than others. For example, several studies involving patients with melanoma have shown an association between the occurrence of cutaneous irAE, such as dermatitis, psoriasis, lichen planus, bullous autoimmune disease, or vitiligo and clinical outcome.^11–13^ Interestingly, patients with melanoma are at higher risk of developing such cutaneous irAEs than patients with other solid malignancies.^14^ This observation suggests that cutaneous irAE, such as vitiligo-like depigmentation of the skin, which is driven by antigen-specific T-cell destruction of cells that carry melanocytic antigens such as gp100, MART-1 or tyrosinase-related proteins 1/2, might be directly related to antigen-specific immunity and may therefore be more strongly correlated with antitumor efficacy than other irAE.^15^ One specific type of vitiligo-like cutaneous irAE is the depigmentation of hair, known as poliosis. Acquired poliosis can appear on the scalp, the eyebrows, the eyelashes or the entire body hair and has been attributed to an immune-response triggered by the cross-reaction of melanoma cells and follicular melanocytes.^16^ Due to the immune privilege of hair follicles poliosis is a rare event and so far, poliosis has only been documented in case reports not suited to assess the significance of poliosis as a clinically accessible marker for treatment outcome.^17–19^

In this multicenter, real-world cohort study, we systematically analyzed patients diagnosed with non-resectable stage III/IV melanoma who received ICB therapy and who developed cutaneous AE to evaluate the role of vitiligo-like depigmentation of the skin or hair on ICB outcomes.

## Methods

### Study design and data source

Patients with advanced, non-resectable American Joint Committee on Cancer (AJCC) stage III/IV melanoma who received ICB therapy, either combined ipilimumab+nivolumab (cICB) or anti-programmed cell death protein 1 (PD-1) monotherapy (nivolumab or pembrolizumab) for at least 1 month were identified from the Skin Cancer Center of the University Medical Center Mainz (discovery cohort). Data were complemented through patients identified from University Medical Centers Frankfurt, Hamburg, Magdeburg, Tübingen and Lübeck (validation cohort). Patients were eligible for analysis if they received ICB therapy for at least 1 month, had a follow-up (FU) of at least 3 months and sufficient documentation on the occurrence of (cutaneous) AEs, including poliosis, vitiligo or other cutaneous irAE >CTCAE grade 2 (Consolidated Standards of Reporting Trials (CONSORT) diagram online supplemental figure 1). Details on treatment and outcome specifics were recorded in an unidentifiable, pseudonymized form at the patient level. Clinical decisions regarding ICB prescription were made independently of this study.

### Patient cohort and study endpoints

At data request (December 2025), we identified 741 patients with non-resectable stage III/IV melanoma treated with ICB regimens and follow-up of at least 3 months. Thereof, 499 patients developed at least one adverse event of CTCAE grade 2 or higher. For subsequent analysis, we focused on patients who developed cutaneous AE of CTCAE grade 2 or higher following ICB initiation (n=221). Cutaneous irAE included pruritus, rash, eczema, psoriasis-like eczemas, bullous pemphigoid, vasculitis, lichen planus, scleroderma, vitiligo and poliosis. We defined the occurrence of poliosis during ICB as an acquired, white discoloration that involved either >50% of the scalp or body hair or complete depigmentation of eyebrows and eyelashes. Patients with vitiligo were suitable for analysis if depigmentation occurred during treatment and involved ≥3% body surface area. Clinical data on baseline patient and tumor characteristics, as well as ICB treatment specifics, AEs classified according to CTCAE criteria, the time, pattern and management of such AE, as well as survival outcomes were collected. Details on AE specifics were used to stratify patients into those with non-cutaneous AE, cutaneous AE and poliosis. Study endpoints were progression-free survival (PFS), objective response rate (ORR) and overall survival (OS).

### Statistical analysis

Descriptive statistics were employed to analyze baseline patient characteristics. Testing for equity between subgroups of patients with/without cutaneous AE was performed using the Mann-Whitney test and Fisher’s exact test. For categorical variables, 95% CIs were calculated using the Clopper-Pearson method. PFS was calculated from the start of ICB therapy until first tumor progression or death from any cause. New primary melanomas were not considered a tumor progress. Best overall response (BOR) was classified according to Response Evaluation Criteria in Solid Tumors (RECIST) V.1.1 criteria. ORR was defined as partial response (PR) or complete response (CR) versus stable disease (SD) or progressive disease (PD) according to standard RECIST criteria. Disease control was defined as SD, PR or CR versus PD. OS was calculated as the time from ICB treatment start to death from any cause. Patients who did not meet the endpoint were censored at the date of last FU. We employed Kaplan-Meier survival plots to illustrate median survival probabilities and to assess clinical associations between the different cutaneous AE groups and survival outcomes. Survival curves were compared using the log-rank test. Median duration of FU was calculated using the reverse Kaplan-Meier method.^20^ Univariate and multivariate Cox regression analyses were used to assess the impact of specific clinical characteristics on OS and PFS. Multivariable analysis was calculated for significant variables by the univariate test or by a priori selection for biological relevance to evaluate their conjoint, independent effects on PFS. HRs with 95% CIs were used to quantify the effect on OS and PFS. To assess whether the favorable impact of poliosis on PFS was consistently maintained across both the discovery and validation cohorts, we used a multistep modeling and meta-analytical approach.^21^ First, Cox regression models with and without an interaction term (*Cut.AE.type x Cohort*) were evaluated and a global interaction test via analysis of variance analysis was performed to determine the prognostic effect of poliosis between both cohort settings. We extracted the HR and standard errors to compute a fixed-effects meta-analysis (*rma*), which allowed us to calculate an overall pooled effect size. Cochran’s Q statistic and the I^2^ metric were used to evaluate statistical heterogeneity and quantify the proportion of total variation across cohorts attributable to true heterogeneity rather than random sampling chance. To account for immortal time bias, we further conducted landmark analysis and used restricted mean survival time analysis to assess statistical significance of survival results exceeding the median FU period using the R-package *survRM2*. Sensitivity analysis was performed using univariable Cox regression for the depicted time points. In all cases, two-tailed p values were calculated and considered significant at p<0.05. SPSS (V.27, IBM, GER) and RStudio (V.4.4.3) were used for all analyses.

## Results

### Patient characteristics

Data were extracted from a total of 741 eligible patients who received ICB therapy between April 2012 and August 2025 for non-resectable stage III/IV melanoma and a FU of at least 3 months at the Skin Cancer Centers of the University Hospital Mainz, Frankfurt, Hamburg, Magdeburg, Tübingen and Lübeck, Germany. Among this cohort, we identified 221 patients who developed any cutaneous irAE of CTCAE grade 2 or higher, including rash, exanthema, eczemas, lichen planus, bullous pemphigoid (n=140) or vitiligo-like leukoderma without hair involvement (n=52) or depigmentation of the hair (n=29) (online supplemental figure 1). Baseline characteristics of this cohort are summarized in table 1. This cohort was used to analyze primary endpoints, including PFS and ORR to ICB.

**Table 1:** Baseline clinico-pathological features of the investigated cohort

Clinicopathological features	Overall cohort	Poliosis	Other cut.AE	P value
Total number of patients	741	29	192	–
Type of cutaneous AE				–
Exanthema	58	2	56	
Dermatitis	57	0	57	
Vitiligo	69	17	52	
Poliosis	29	29	0	
Bullous pemphigoid	10	0	10	
Psoriasis-like	6	1	5	
Lichen ruber	7	0	7	
Pruritus	3	0	3	
Folliculitis	3	1	2	
Scleroderma	2	0	2	
Vasculitis	3	0	3	
Viral exanthema	2	0	2	
Alopecia areata	1	1	0	
Median age at start of ICB (years, 95% CI)	66.0 (64.5 to 66.5)	59 (51.0 to 64.0)	66 (63.5 to 67.5)	**0.013**
Gender				0.306
Male (%)	453 (61.1)	21 (72.4)	118 (61.5)	
Female (%)	288 (38.9)	8 (27.6)	74 (38.5)	
A Primary tumor characteristics				
Ulceration* (%)	285/524 (54.4)	7/16 (43.8)	69/143 (48.3)	0.767
BRAF mutation status† (%)	293/723 (59.5)	13/29 (44.8)	72/181 (39.8)	0.685
B Baseline patient characteristics and ICB treatment details				
LDH >245 U/mL at baseline‡ (%)	190/321 (59.2)	16/22 (55.2)	91/180 (50.6)	0.692
AJCC stage at baseline				–
IIIB (%)	19	2 (6.8)	12 (6.3)	
IIIC (%)	76	5 (17.2)	48 (25)	
IIID (%)	7	1 (3.4)	5 (2.6)	
IV (%)	639	21 (72.4)	127 (66.1)	
Multifocal disease (%)	313/726 (43.1)	17/22 (58.6)	97/178 (54.5)	0.841
Melanoma brain metastasis (%)	195/740 (26.4)	9/28 (32.1)	43/192 (22.4)	0.337
Treatment-line of ICB				1
First (%)	643 (86.8)	26/29 (90.9)	171 (89.1)	
Second (%)	98 (13.2)	3 (9.1)	21 (10.9)	
ICB agent				0.255
IPI+NIVO (%)	480 (64.8)	23 (79.3)	121 (63.0)	
Anti-PD-1 (%)	239 (32.3)	6 (20.7)	68 (35.4)	
Ipilimumab (%)	22 (3)	–	3 (1.6)	
Median duration of ICB (95% CI)	4 months (3.5 to 8)	14 months (11 to 22.5)	8 months (10.5 to 15.5)	0.136
Best overall response (RECIST V.1.1)§				**<0.001**
CR (%)	116 (16.5)	19 (65.5)	42 (22.2)	
PR (%)	141 (20)	8 (27.6)	40 (21.6)	
SD (%)	160 (22.7)	2 (6.9)	37 (20)	
PD (%)	287 (40.8)	–	66 (35.7)	
Eventual tumor progression on ICB (%)	489 (66)	5 (17.2)	114 (59.4)	**<0.001**
Reasons for ICB cessation¶ (%)				–
Disease progression	320/589 (54)	1/19 (4.5)	27/62 (43.5)	
Toxicity	119/589 (20)	7/19 (36.8)	17/62 (27.4)	
Completion of 24 months	60/589 (10)	6/19 (31.6)	7/62 (11.3)	
Patient preference	29/589 (5)	0	7/62 (11.3)	
Ongoing	61/589 (10)	6/19 (31.6)	4/62 (6.5)	
Median progression-free survival (95% CI)	7 months (6 to 9)	NR	14 months (9 to 31)	**<0.001**
C Survival and follow-up				
Median follow-up on start of ICB therapy (95% CI)	37 months (33 to 43)	48 months (38 to 61)	51 months (42 to 61)	0.19
Median overall survival (95% CI)	54 months (43 to 83)	NR	83 (57 to NR)	**<0.001**
Restricted mean survival time (95% CI) at 48 months landmark	–	47.2 months (45.5 to 48.8)	37.7 months (35.4 to 40.0)	**<0.001**
3-year OS rate (95% CI)	57.3% (53.4% to 61.6%)	96% (88.6% to 100%)	69.4% (62.7% to 76.8%)	–
Deceased (%)	303 (40.9)	1 (3.4)	71 (37)	**<0.001**

Statistically significant differences of features between poliosis and other.cut.AE groups are denoted in bold (p<0.05)

* Primary tumor ulceration status was evaluable for 524 patients.

† BRAF mutation status was available in 723 patients.

‡ LDH levels at baseline were available in 321 patients.

§ Data on treatment response was available in 704 patients.

¶ Data on reasons for treatment cessation was available for 589 patients.

AE, adverse event; AJCC, American Joint Committee on Cancer; AJCC, American Joint Committee on Cancer; BRAF, B-raf gene; CR, complete response; ICB, immune checkpoint blockade; IPI, Ipilimumab; LDH, Lactate Dehydrogenase; NIVO, Nivolumab; NR, not reached; OS, overall survival; PD, progressive disease; PD-1, programmed cell death protein 1; PR, partial response; RECIST, Response Evaluation Criteria in Solid Tumors; SD, stable disease.

Patients who developed poliosis were predominantly male (72.4%) and had a median age of 59 years at ICB start. Ulceration of the primary tumor, a positive BRAF mutation status and tumor stage at baseline were equally distributed between patients with or without poliosis. Patients who were treated after 2018 were staged according to the 2018 (Eighth Edition) AJCC melanoma staging criteria, while patients who received prior ICB were reclassified according to AJCC eighth criteria. Patients developing poliosis tended towards elevated Lactate dehydrogenase (LDH) levels at baseline (55.2% vs 50.6%), more often presented with bulky disease involving more than two organ sites of distant metastases (58.6% vs 54.5%) or melanoma brain metastases (32.1% vs 22.4%, p*=*0.34) compared with patients who developed other cutaneous AE, although these trends were below statistical significance.

ICB regimens were predominantly administered in a first-line setting (86.8%) and included ipilimumab+nivolumab (cICB) (64.8%), anti-PD-1 monotherapy (32.2%) or ipilimumab monotherapy (3%) with no significant differences between both cutaneous AE subgroups. While the median duration of ICB treatment was 4 months in the overall cohort; there was a trend towards longer ICB treatments in the poliosis subgroup (14 vs 8 months), albeit below statistical significance. In the overall cohort, 528 patients prematurely discontinued ICB treatments mostly due to tumor progression (54%) and toxicity (20%). Similarly, patients who developed cutaneous adverse events (AEs) other than poliosis predominantly discontinued treatment due to tumor recurrence or toxicity. In contrast, most patients with poliosis either remained on ICB therapy at data cut-off, completed the full 24-month regimen prior to discontinuation (31.6%), or stopped due to toxicity (36.8%).

In the overall patient cohort, 257 patients achieved an objective response (36.5%), while for patients developing poliosis 27/29 (93.1%) patients showed an objective response. In the overall patient cohort, 489 patients (66%) eventually developed a tumor recurrence with a median PFS of 7 months (95% CI 6 to 9 months). Here, patients with an onset of poliosis on ICB treatment recurred less often (17.2 vs 59.4%, p<0.001) resulting in a prolonged PFS (not reached (NR) vs 14 months, p*<*0.001) compared with patients developing other cutaneous AEs. At data cut-off, median OS was 54 months (43–83 months), with all but one patient alive among those developing poliosis, whereas 71 patients (37%) of patients developing other cutaneous irAEs deceased.

### Patients with cutaneous AE present with favorable survival outcomes on ICB therapy

To dissect the role of cutaneous irAE in general and vitiligo-like depigmentation of the hair and skin in particular, we next analyzed the association of such irAE with primary endpoints. We observed that patients who developed any kind of irAE of CTCAE grade 2 or above presented with slightly better PFS following ICB therapy (9 vs 5 months, p*<*0.001; Figure 2A) than patients without documented irAE of grade 2 or higher. More notably, we observed that patients who developed cutaneous irAE of grade 2 or above showed a significantly longer PFS compared with patients with non-cutaneous irAE or patients without documented irAE >grade 2 (median PFS: 24 vs 6 vs 5 months, p*<*0.001; Figure 2B). These results were complemented by univariable Cox-regression analysis, that confirmed a significant association of cutaneous irAE, ICB treatment line, and BOR on PFS outcomes (online supplemental table S1). This link between the presence of cutaneous AE and PFS was also confirmed in multivariate Cox regression analysis (HR for no AE vs cutaneous AE: 2.96, p*<*0.001) (table 2).

**Figure 2:**
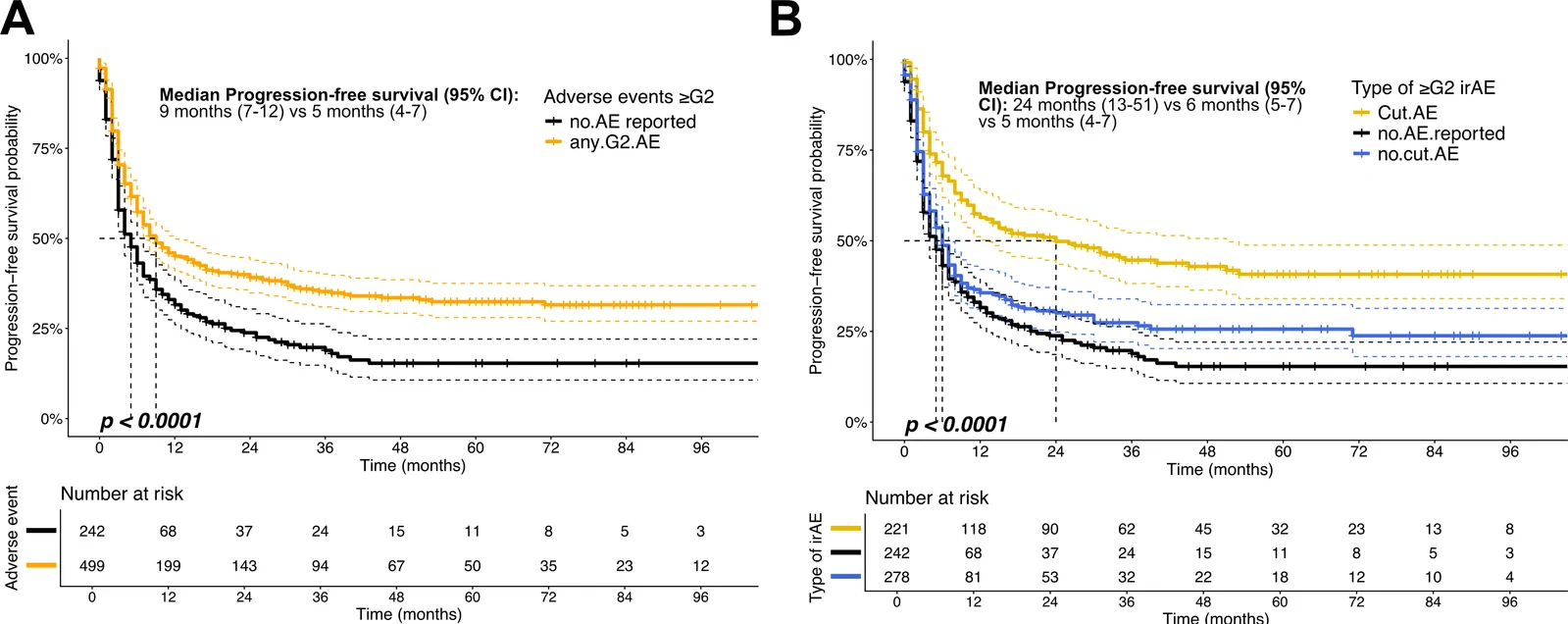
Patients with documented cutaneous adverse events present with favorable progression-free survival following immune-checkpoint blockade therapy. (**A**) Kaplan-Meier plot depicting progression-free survival probabilities for patients who did not develop any adverse events of Common Terminology Criteria for Adverse Events (CTCAE) grade 2 or above or who did develop any AE of CTCAE grade 2 or above. Median PFS is marked by black dotted lines, and 95% CI of the investigated groups are indicated by color-coded dotted lines. Numbers at risk are provided at the bottom panel. Statistical significance was calculated using an unadjusted log-rank test. (**B**) Progression-free survival probabilities for patients who either developed no cutaneous adverse event of CTCAE grade 2 or above, who had no documented adverse events or who developed cutaneous adverse events of CTCAE grade 2 or higher. Median PFS is marked by black dotted lines and 95% CI of the investigated groups are indicated by color-coded dotted lines. Numbers at risk are provided at the bottom panel. Statistical significance was calculated using an unadjusted log-rank test. Groupwise comparisons revealed a statistically significant PFS prolongation in patients developing cutaneous irAE compared with patients without irAE (p*<*0.001) or non-cutaneous irAE (p*<*0.001). AE, adverse event; irAE, immune-related adverse event; PFS, progression-free survival.

**Table 2:** Multivariate Cox regression analysis for progression-free survival of the overall patient cohort (n=741)

Variables	Subgroups	HR	95% CI	P value
Age (years)	>65 vs ≤65	0.95	0.7 to 1.3	0.719
Gender	Male vs female	1.1	0.8 to 1.5	0.589
LDH elevated	Yes vs no	1.72	1.24 to 2	**0.001**
Multifocal disease	Yes vs no	1.56	1.13 to 2.1	**0.007**
ICB line	Second vs first	1.39	0.97 to 2.0	0.071
Adverse events during ICB	Cut.AE (ref) vs	Ref	Ref	
	no.AE.reported vs	2.96	2.04 to 4.3	**<0.001**
	extracutaneous.AE	1.78	1.12 to 2.8	**0.015**

Statistically significant associations with progression-free survival are denoted in bold (p<0.05)

CTCAE, Common Terminology Criteria for Adverse Events; Cut.AE, cutaneous adverse events of at least CTCAE grade 2; ICB, immune checkpoint blockade; LDH, Lactate dehydrogenase; Non.Cut.AE, adverse events affecting other organs than the skin/hair.

### Poliosis onset during ICB is linked to improved ICB response and survival that is distinct from other cutaneous adverse events

Given the notable association between cutaneous irAE and treatment outcomes, we next dichotomized cutaneous AE into poliosis or other cutaneous irAE: Thereby, we found that patients with an onset of poliosis during ICB presented with a significantly longer PFS (median PFS not reached at a median FU of 48 months) compared with all other cutaneous AE (median PFS: 14 months, p*<*0.001; Figure 3A) and OS (NR vs 83 months, p*<*0.001; Figure 3B; restricted mean survival at 48 months: 47.2 months vs 37.7 months, p<0.001). To distinguish the role of poliosis from the previously described association of vitiligo for ICB treatment outcomes, we further separated patients with vitiligo-like depigmentation of the skin from patients developing other cutaneous irAE. In doing so, we observed that patients with an onset of poliosis during ICB presented with a significantly improved PFS (NR vs 31 vs 9 months, p*=*0.001; Figure 3C) and ICB-response rates (93.1% vs 58.8% vs 38.8%, p*<*0.001; Figure 3D, online supplemental figure 2A) compared with patients developing vitiligo of the skin only. Our results further showed that all but one patient with an onset of poliosis were still alive at a median FU of 48 months (median OS: NR vs NR months for vitiligo of the skin only and 69 months for other cutaneous irAE, p*<*0.001; Figure 3E, (online supplemental figure 2B).

**Figure 3:**
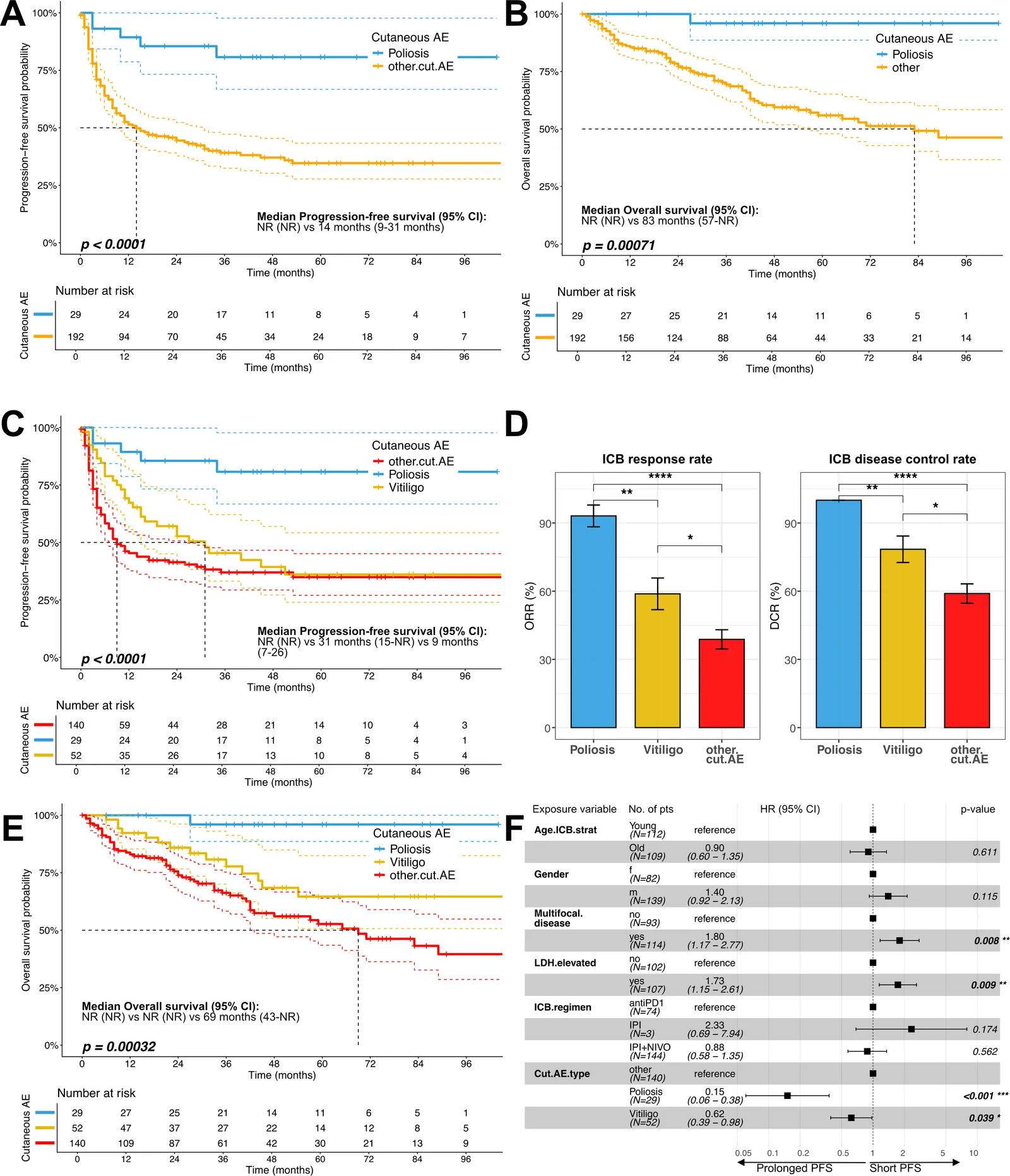
Patients developing poliosis during immune-checkpoint blockade are characterized by improved response and progression-free survival. (**A**) Kaplan-Meier plot depicting progression-free survival probabilities for patients who either developed poliosis or any other cutaneous adverse event during ICB. Median PFS is marked by black dotted lines and 95% CI of the investigated groups are indicated by color-coded dotted lines. Numbers at risk are provided at the bottom panel. Statistical significance was calculated using an unadjusted log-rank test. (**B**) Overall survival probabilities for patients who either developed poliosis or any other cutaneous adverse event during ICB. Median OS is marked by black dotted lines and 95% CI of the investigated groups are indicated by color-coded dotted lines. Numbers at risk are provided at the bottom panel. Statistical significance was calculated using an unadjusted log-rank test. (**C**) Kaplan-Meier plot depicting progression-free survival probabilities for patients who either developed poliosis, vitiligo or any other cutaneous adverse event during ICB. Median PFS is marked by black dotted lines and 95% CI of the investigated groups are indicated by color-coded dotted lines. Numbers at risk are provided at the bottom panel. Statistical significance was calculated using an unadjusted log-rank test. In groupwise comparisons, poliosis showed a significantly prolonged PFS compared with vitiligo (p*=*0.001) and other cutaneous irAE (p*<*0.001). (**D**) Bar charts summarizing mean objective response rates (left panel) or disease control rates (right panel) following ICB initiation by type of cutaneous adverse event. Error bars indicate SEs of the mean. Mean ORR (95% CI) was 93.1% (83.7% to 100%) versus 58.8% (45.3% to 72.3%) versus 38.8% (30.7% to 46.9%), while disease-control rates were 100% versus 78.4% (67.1% to 89.7%) versus 59.0% (50.8% to 67.1%). Statistical significance was calculated using Fisher’s exact test. (**E**) Kaplan-Meier plot depicting overall survival probabilities for patients who either developed poliosis, vitiligo or any other cutaneous adverse event during ICB. Median OS is marked by black dotted lines and 95% CI of the investigated groups are indicated by color-coded dotted lines. Numbers at risk are provided at the bottom panel. Statistical significance was calculated using an unadjusted log-rank test. (**F**) Forest plot illustrating results from multivariate Cox regression analysis for progression-free survival, including HRs for selected clinical exposure variables. AE, adverse event; ICB, immune checkpoint blockade; irAE, immune-related adverse event; NR, not reached; OS, overall survival; ORR, objective response rate; PFS, progression-free survival.

These results were confirmed in both univariable (online supplemental figure 2C, online supplemental table S2) and multivariate Cox regression analysis for PFS (figure 3F), as well as OS (online supplemental figure 2D), suggesting that the prognostic relevance of poliosis was independent of ICB regimen, metastatic site, baseline LDH levels and patient demographics. Indeed, we observed that patients developing poliosis retained a survival advantage even in the presence of adverse prognostic factors such as multifocal disease, melanoma brain metastases or premature ICB discontinuation, which were significantly linked to short PFS in patients with other cutaneous AE (online supplemental figure 2E).

Given the multicenter character of this study, we next validated whether the favorable prognostic impact of poliosis onset was found across both discovery and validation cohorts (figure 4A, online supplemental table S3). To this end, we used multistep modeling and a fixed-effect model, which confirmed a consistent link of poliosis onset with prolonged PFS (HR: 0.17, p=0.0026) across both discovery and validation cohort that was not attributable to inter-cohort variance (I^2^=0.0%, p*=*0.61) (figure 4B).

**Figure 4:**
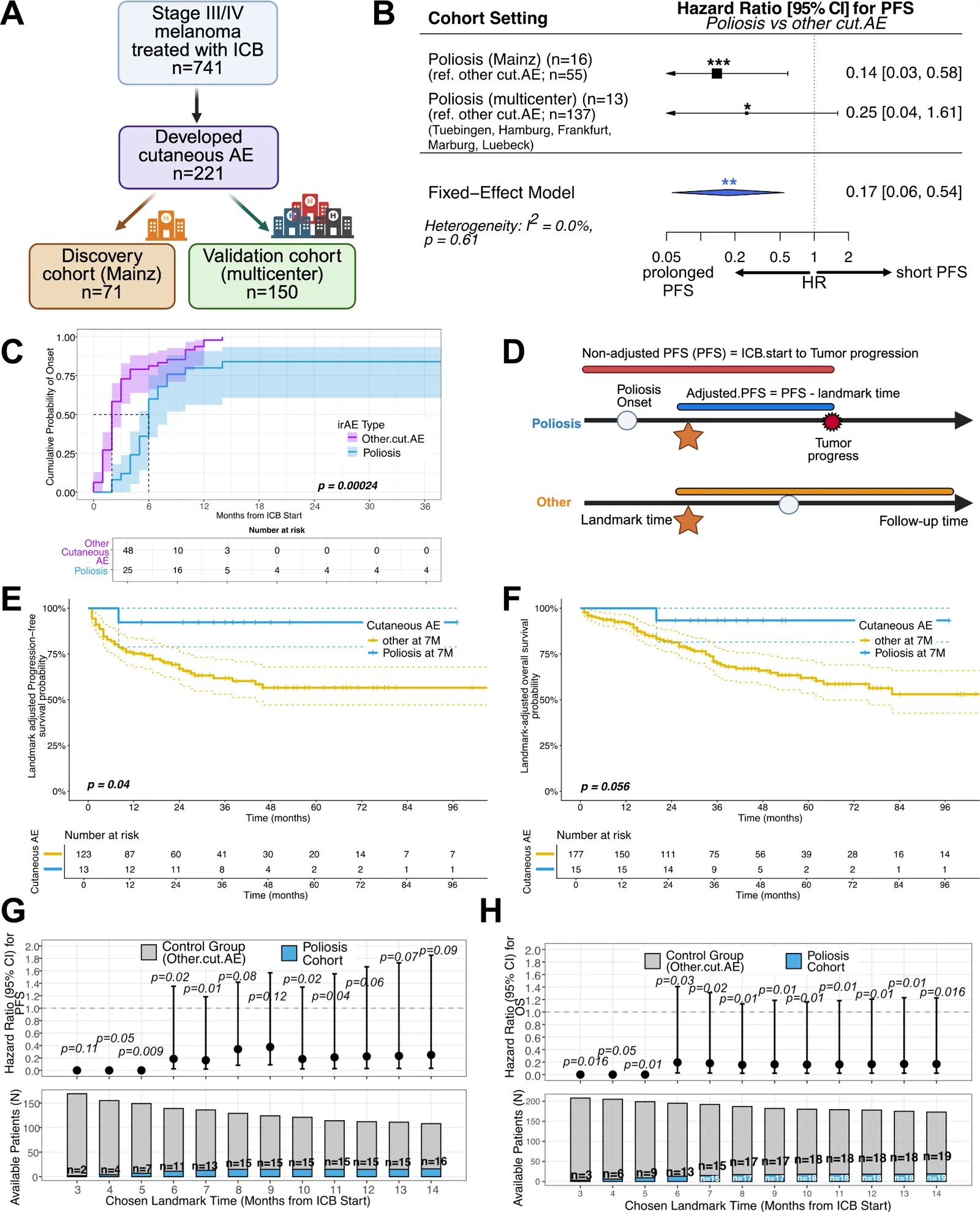
Poliosis associates with prolonged survival in external multicenter validation and time-dependent landmark analysis. (**A**) Graphical summary of the analytical approach used to study the impact of poliosis across skin cancer centers. (**B**) Forest plot depicting results from interaction analysis that separates the individual HRs for progression-free survival in patients developing poliosis within the discovery cohort (Mainz, n=16) and validation cohorts (multicenter, n=13). A fixed-effect model was used to assess whether the protective effect of poliosis was consistent across both cohorts (pooled HR: 0.17). Heterogeneity diagnostics were run to assess variability across both cohorts (0.0%). (**C**) Cumulative incidence plot summarizing the median time until development of poliosis or other cutaneous AEs. Median time to event is marked by black dotted lines and 95% CI of the investigated groups are indicated by color-coded dotted lines. Numbers at risk are provided at the bottom panel. Statistical significance was calculated using an unadjusted log-rank test. Log-rank test showed a significantly prolonged time for patients to develop poliosis (6 months, 95% CI: 5 to 8) when compared with the time to develop other cutaneous AE (median: 2 months; 2 to 3). (**D**) Schematic to summarize the analytical approach to conduct landmark-adjusted survival analysis. (**E**) Kaplan-Meier plot depicting landmark-adjusted progression-free survival probabilities for patients who either developed poliosis at landmark=7 months or those patients with cutaneous adverse events who did not develop poliosis by the landmark time. Patients who had negative PFS values on adjusted landmark computation were omitted from the analysis. Median landmark-adjusted PFS is marked by black dotted lines and 95% CI of the investigated groups are indicated by color-coded dotted lines. Numbers at risk are provided at the bottom panel. Statistical significance was calculated using an unadjusted log-rank test. (**F**) Kaplan-Meier plot depicting landmark-adjusted overall survival probabilities for patients who either developed poliosis at landmark=7 months or those patients with cutaneous adverse events who did not develop poliosis by the landmark time. Patients who had negative OS values on adjusted landmark computation were omitted from the analysis. Median landmark-adjusted OS is marked by black dotted lines and 95% CI intervals of the investigated groups are indicated by color-coded dotted lines. Numbers at risk are provided at the bottom panel. Statistical significance was calculated using an unadjusted log-rank test. (**G**) Sensitivity analysis presented as an inverted forest plot (top) with results from univariate Cox regression analysis for progression-free survival, including HRs with 95% CI and corresponding p values for patients who did or did not develop poliosis by the specified landmark time. On the bottom stacked bar charts summarize the number of patients included in the analysis at each landmark time point who were either counted within the poliosis group or those patients with cutaneous AE who had not developed poliosis by the landmark time or for which landmark adjusted PFS was smaller than 0 months. (**H**) Sensitivity analysis presented as an inverted forest plot (top) with results from univariate Cox regression analysis for progression-free survival including HRs with 95% CI and corresponding p values for patients who did or did not develop poliosis by the specified landmark time. On the bottom stacked bar charts summarize the number of patients included in the analysis at each landmark time point who were either counted within the poliosis group or those patients with cutaneous AE who had not developed poliosis by the landmark time or for which landmark adjusted PFS was smaller than 0 months. AE, adverse event; ICB, immune checkpoint blockade; irAE, immune-related adverse event; OS, overall survival; PFS, progression-free survival.

Due to the late onset of poliosis relative to other cutaneous AE (median time to poliosis: 6 months vs 2 months, p*<*0.001; Figure 4C), we further complemented our analysis through the addition of landmark analysis to account for “immortal time bias”: Using the median time to poliosis as initial landmark (>6 months) (figure 4D), our results confirmed a significant association of poliosis onset with landmark-adjusted PFS (figure 4E) and a trend towards prolonged landmark-adjusted OS (figure 4F), which was largely recapitulated in a time-dependent landmark-adjusted sensitivity analysis for PFS (figure 4G) and OS (figure 4H).

### Poliosis is a late irAE during ICB therapy that occurs independent of treatment duration marking durable tumor control

Given the relevance of poliosis for ICB treatment outcomes we finally characterized these patients in more detail: We observed that poliosis patients developed a mean of 2.5 other irAE (table 3). These irAE included other cutaneous AE such as vitiligo but also colitis, pneumonitis, hepatitis and nephritis that usually occurred prior to poliosis onset at a median of 2 months after ICB initiation. Notably, there was a substantial overlap in the occurrence of poliosis and vitiligo following ICB start (n=17; online supplemental figure 3A), which resulted in a strong correlation between both factors on the overall cohort level (R=0.23, p*<*0.001, online supplemental figure 3B,C), whereas this correlation was less strong when compared with any other cutaneous AEs. Hence, we further analyzed whether the favorable prognostic outcomes observed for all patients developing poliosis was maintained after categorizing such patients who developed both poliosis and vitiligo (n=17) into the group of vitiligo patients or even treating them as a separate group altogether: In fact, we found that the association between the occurrence of poliosis without presence of vitiligo correlated even stronger with PFS and OS (online supplemental figure 3D–G) and that this favorable association was maintained on multivariate Cox regression (online supplemental figure 3H,I).

**Table 3:** Details on irAE during or 3 months upon ICB cessation in the cohort of patients who developed poliosis (n=29)

Features	Poliosis
Median time to first response	4.5 months
Median time to first AE (95% CI)	2 months (2 to 7 months)
Development of other AEs	Exanthema (5/29) Vitiligo (17/29) Hypophysitis (3/29) Thyroiditis (4/29) Pneumonitis (4/29) Colitis (6/29) Nephritis (4/29) Hepatitis (9/29)
Mean number of other AEs (95% CI)	2.5 (2.1 to 2.9)
Mean maximum toxicity grade of other AEs according to CTCAE (95% CI)	2.4 (2.1 to 2.6)
Systemic steroid treatments during ICB	11/18
Median time to first signs of poliosis (95% CI)	6 months (5.5 to 10)

AE, adverse event; CTCAE, Common Terminology Criteria for Adverse Events; ICB, immune checkpoint blockade; irAE, immune-related AE.

Despite the high incidence of irAE in patients developing poliosis, irAE were in most cases moderate in severity. However, in 11/18 cases irAEs required the initiation of immunosuppressive treatments and led to premature treatment cessation in seven patients (table 1). Among these seven patients, four patients received only five cycles of either cICB or anti-PD-1 monotherapy for achieving durable remissions of at least 30 months (online supplemental figure 4A), suggesting that prolonged ICB exposure might not be required for durable tumor responses in poliosis patients.

Critically, tumor remissions were observed across any organ site, including pulmonal, hepatic, abdominal and cerebral lesions (online supplemental figure 4B,C), indicating that cross-reactivity against melanocytic antigens in hair follicles may evoke strong and durable tumor remissions regardless of the total time of ICB administration and metastatic sites.

## Discussion

The advent of ICB has fundamentally transformed the treatment landscape of patients with advanced melanoma. While durable responses to ICB-therapies are limited to approximately 20% of patients with metastatic melanoma, the clinical success of ICB often coincides with the risk of developing potentially severe irAEs that correlates with the duration of ICB regimens.^22 23^ Given the lack of compelling evidence to support personalized ICB treatment duration, a pivotal clinical challenge is to identify clinically accessible markers that reliably indicate that a response is sufficiently profound and durable to persist after treatment cessation.

In this study, we investigated a comprehensively annotated, multicenter cohort of 741 patients receiving ICB for non-resectable stage III/IV melanoma, which allowed us to dissect the link between cutaneous irAE and particularly vitiligo-like depigmentation of the hair or skin for ICB efficacy.

Here, prior reports indicate that the occurrence of irAE provides evidence for an effective immune activation.^23 24^ However, whether such non-specific activation is linked to improved clinical outcomes remains controversial.^22^ Previous works rather suggest that certain irAEs may be more related to antitumor efficacy than others. Specifically, some cutaneous irAE such as vitiligo-like depigmentation showed a stronger association with antitumor efficacy compared with other irAE. Given that vitiligo occurs more frequently in patients receiving ICB for melanoma compared with other solid tumors,^25^ these findings implicate a direct link between the occurrence of vitiligo and antigen-specific immunity,^26 27^ where depigmentation of the skin represents a bystander effect of an antigen-specific immune responses against melanocytic antigens.

Our results recapitulate these observations as both the occurrence of cutaneous irAE in general and vitiligo in particular showed a significant association with ICB response and survival outcomes compared with other non-cutaneous irAE. Importantly, these favorable outcomes were not driven by patient demographics, baseline tumor burden or treatment regimen in multivariate analysis. These clinical observations reflect on the immunological mechanisms underlying ICB-induced vitiligo, that is the result of T-cell driven destruction of epidermal melanocytes and melanoma cells. Here, previous studies identified a set of shared biomarkers linked to ICB benefit and the development of vitiligo,^28^ that involved an interferon (IFN)-γ signature including elevated expression of CXCL9/10, IDO1 and STAT1.^29–31^ Furthermore, T-cell receptor sequencing data demonstrated that T-cell clones in vitiligo lesions were distinct from those in paired primary melanomas but shared with metastatic tissues, indicating a de novo immune response linking tumor and melanocyte destruction.^32^

We further show that depigmentation of the hair (poliosis) represents an irAE linked to substantial clinical benefit that was distinct from other cutaneous irAE including vitiligo-like depigmentation of the skin. This clinical benefit was apparent in terms of enhanced ICB response, prolonged PFS and most importantly the advent of durable remissions. This association was observed regardless of initial tumor burden and total ICB duration, suggesting poliosis might constitute a strong signal of profound antigen-specific tumor control. Given the fact that patients developing poliosis tended towards elevated LDH levels at baseline, more often presented with bulky disease involving more than two organ sites of distant metastases or melanoma brain metastases compared with patients who developed other cutaneous AE, the prognostic value of poliosis is even more striking. Interestingly, the onset of poliosis was more frequently observed for combination immunotherapy regimens, suggesting that a stronger level of immune activation might be required for the development of poliosis. While poliosis was detected only in a minor fraction of ICB-treated patients (<3%) and has so far only been reported in small case reports,^17 19 33 34^ the clinical observations are immunologically well founded: Depigmentation of the hair is a result of T-cell driven destruction of melanocytic stem cells (MSCs) that are located in the hair bulb. As opposed to fully differentiated epidermal melanocytes, that are targeted in autoimmune vitiligo, these MSC have an immune-privileged status. This immune privileged status is maintained through low expression of major histocompatibility complex class I and II, local immunosuppressive cytokines, the presence of regulatory T cells and Fas ligand expression limiting effector T-cell recruitment and function.^35–38^ Meanwhile, MSC retain low expression of melanocyte differentiation antigens such as MART-1, gp100 and tyrosinase that may be recognized by autoreactive T cells in antitumor immune responses to melanoma.^39 40^ In line, it has been reported that skin resident memory T cells (Trm) are critical for perpetuating antitumor immunity in melanoma. These melanoma-antigen-specific Trm were detected specifically in melanocyte-depleted hair follicles and are capable of producing IFN-γ.^41^ The occurrence of poliosis might therefore represent a unique immunological phenotype linked to the presence of Trm that can overcome the immune-privileged microenvironment of follicular melanocytes.

Our study has certain limitations such as the retrospective nature and the relatively short follow-up period for poliosis patients who discontinued ICB regimens. Here, further FU data will be necessary and might aid to the accuracy and the long-term stability of our results. Our study was not randomized and included a small number of patients with distinct irAE; therefore, interpretation of subgroups requires caution. Measurement of response to subsequent therapy was performed by the treating clinician, rather than centralized review and FU imaging was done according to the standards of the different participating centers, which might result in variations in timing of tumor assessment and response evaluation. While poliosis was a reliable marker indicating profound ICB benefit, poliosis onset was a late event on ICB initiation that did usually occur 6 months following initial ICB administration limiting its use as an early-onset response marker.

A key unresolved question is whether the immune response resulting in depigmentation of hair follicles and the tumor response are indeed driven by the same antigen-specific T cells. Addressing this issue will require further experimental validation in preclinical models and longitudinal translational investigations, for which the current results are predicate justification.

While our results detail the link between poliosis and durable ICB response in patients with melanoma, there is a small number of case reports detailing the onset of vitiligo-like depigmentation of the skin and hair in non-melanoma cancers.^42^ Further studies are needed to investigate whether these autoimmune phenomena are also a marker of therapeutic benefit in non-melanoma cancers and whether these result from tumor-specific T-cell responses.

Collectively, our study identified poliosis as a distinct and immunologically informed, clinically accessible marker linked to strong and durable ICB responses. Our findings emphasize that the occurrence of poliosis showed a superior association to ICB response when compared with established clinical markers such as vitiligo and that its prognostic value was independent of initial tumor burden, ICB treatment duration or the presence of brain metastases. As poliosis arises late during treatment, it does however not serve as a predictive biomarker of ICB response, but rather signals that an ongoing response is deep and likely to persist even after treatment cessation. These results will facilitate clinical decision-making on ICB cessation and lay the foundation for translational investigations dedicated to identifying shared melanocyte antigens within hair follicles as novel therapeutic targets to evoke such durable clinical responses.

## Supplementary material

10.1136/jitc-2026-015306 online supplemental file 1

## Data Availability

Data are available upon reasonable request.
