# Supplementary material for: Poliosis predicts durable response to immune-checkpoint blockade in metastatic melanoma: a multicenter cohort study
Source: J Immunother Cancer. 2026 Oct 5;14(10):e015306. doi: 10.1136/jitc-2026-015306 (PMC13647029; doi:10.1136/jitc-2026-015306)
Supplement: Supplementary file 1 [file jitc-14-10-s001.pdf]

Supplemental Figures and Tables

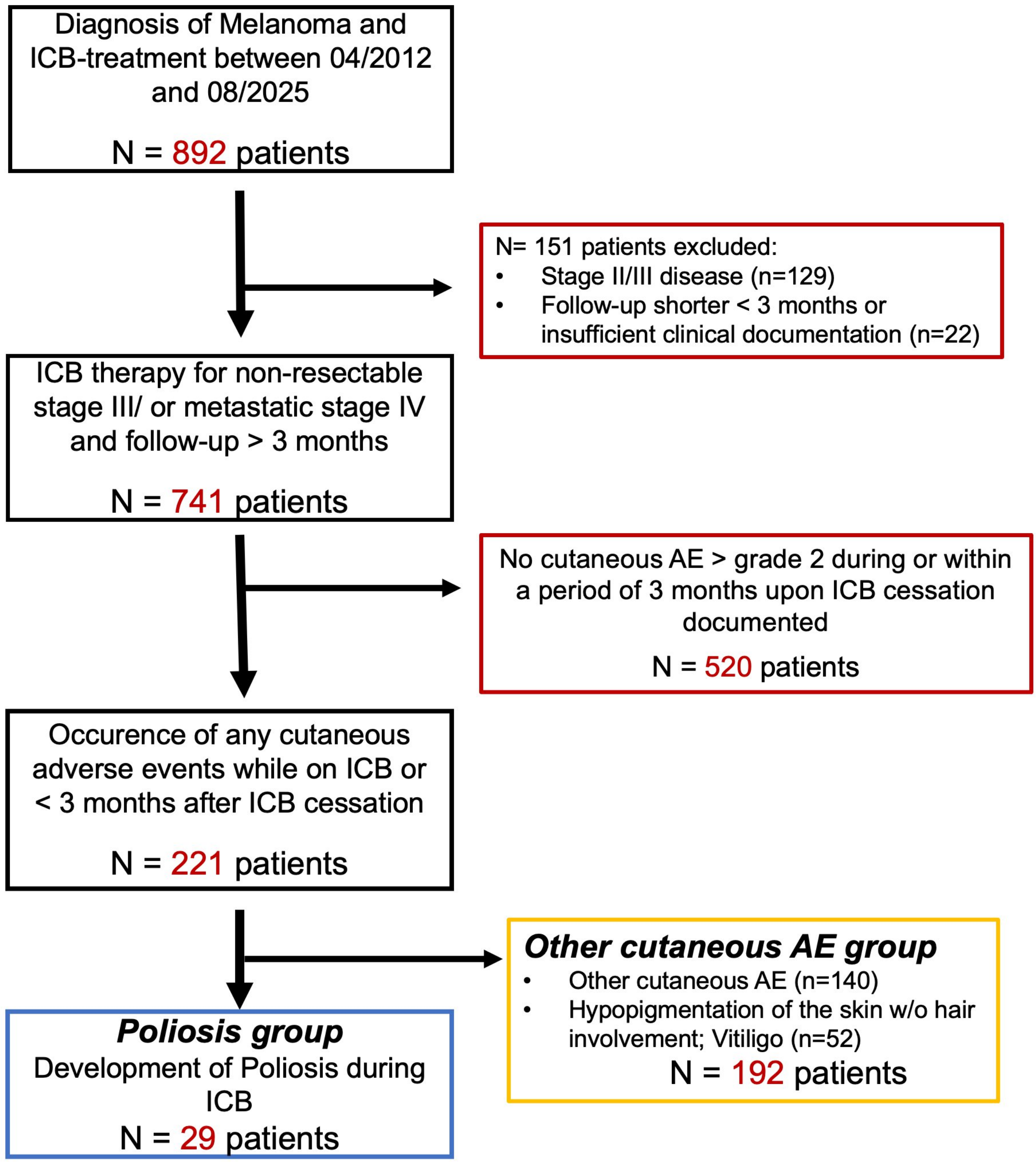

Supplemental Figure 1: CONSORT diagram showing the steps involved in the identification or the investigated ICB-cohort developing poliosis.

From all patients treated with immune-checkpoint blockade agents at the Skin Cancer Centers Mainz, Frankfurt, Hamburg, Magdeburg, Tübingen and Lübeck between 04/2012 and 08/2025 we selected those with non-resectable stage III / IV melanoma and a follow-up of at least 3 months. Within this cohort we identified 221 patients with cutaneous adverse events of grade 2 or above from which we detected 29 patients who specifically developed a whitening of the hair, termed poliosis.

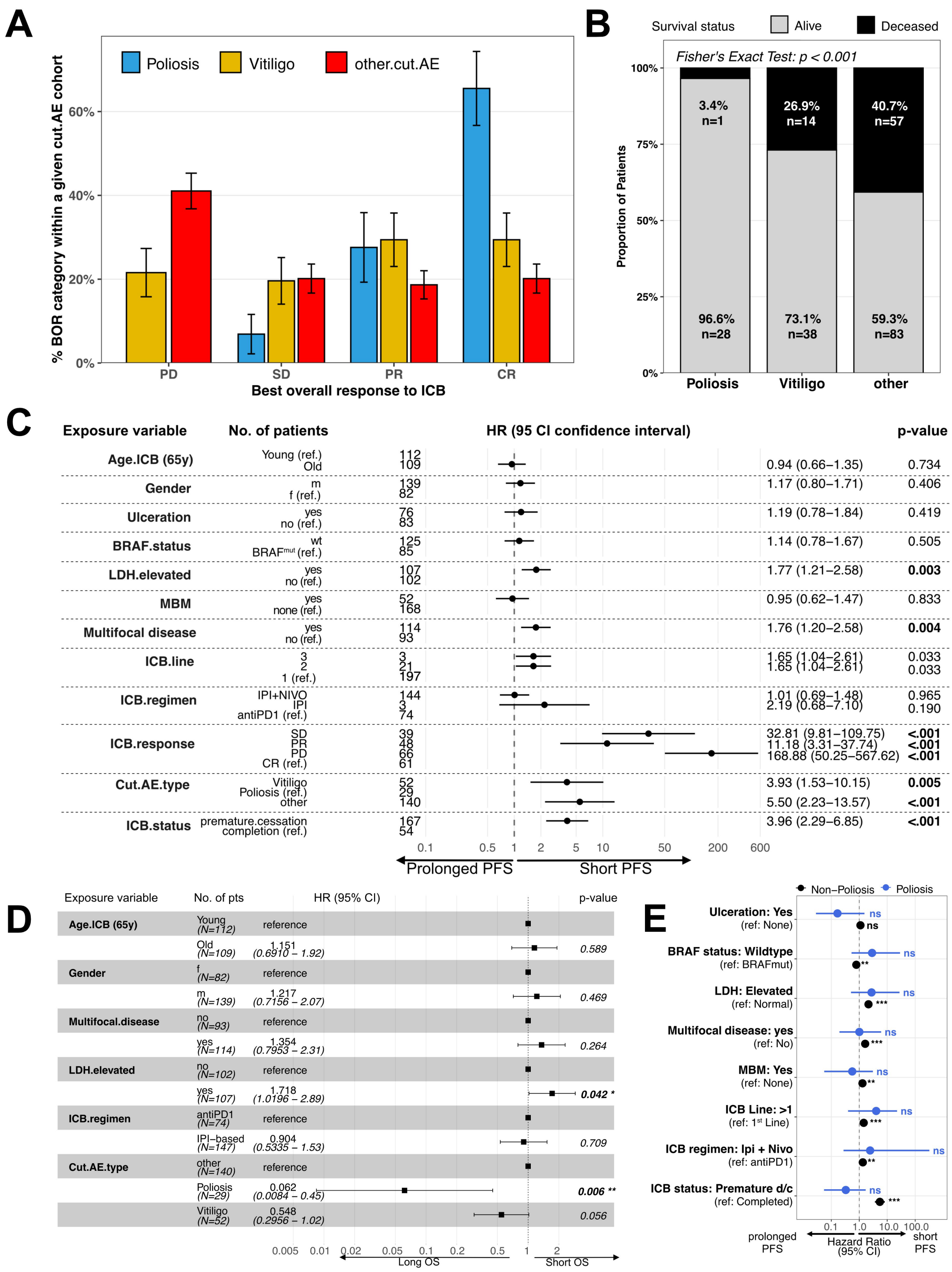

Supplemental Figure 2: Poliosis is an independent predictor of prolonged survival in patients with metastatic melanoma who received immune-checkpoint inhibitors.

(A) Grouped Bar chart depicting the mean proportions of each best overall response variable across patients with cutaneous adverse events stratified by the type of cutaneous AE. Error bars indicate standard error of the mean (SEM).

(B) Stacked barplot summarizing the share of patients who developed cutaneous AE during ICB who were still alive or deceased within the follow-up period stratified by the type of cutaneous AE. Statistical significance was assessed using Fisher´s exact test.

(C) Forest plot depicting results from univariate Cox-regression analysis for progression-free survival of the selected exposure variables with corresponding hazard ratios and number of patients in each group.

(D) Forest plot summarizing results from multivariate Cox regression analysis for overall survival with the corresponding 95% confidence intervals were estimated using multivariate Cox-regression for baseline patient demographics, tumor burden and treatment characteristics as well as cutaneous adverse events.

(E) Grouped forest plot shows results of univariate Cox-regression analysis for progression-free survival between patients who did (n=29) or who did not develop poliosis (n=192) upon ICB start. Tested exposure variables were selected based on prior literature for adverse prognostic features linked to poor ICB responses. Abbreviations: *Abbreviations: ICB = immune-checkpoint blockade; cut.AE = cutaneous adverse event; cICB= combination immune-checkpoint blockade using Ipilimumab 3mg/kg bw + nivolumab 1mg/kg bw; d/c = discontinuation*; *MBM = melanoma brain metastases.*

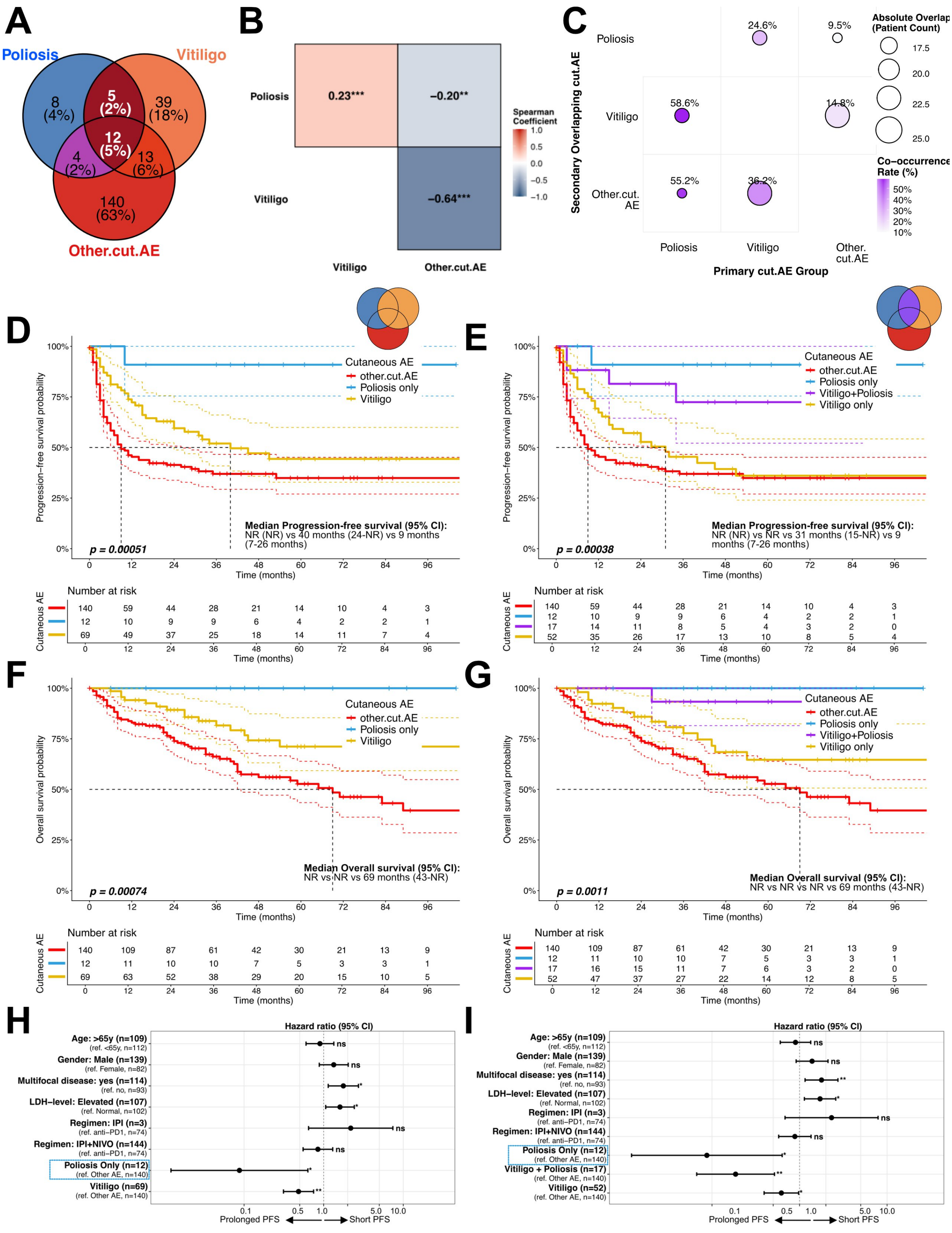

Supplemental Figure 3: Poliosis is a late onset cutaneous adverse event that associates with the occurrence of vitiligo

(A) Venn diagram depicting adverse event counts for Poliosis, Vitiligo and other cutaneous adverse events as well as their co-occurrence following ICB initiation.
(B) Correlation matrix for the co-occurrence of depicted cutaneous adverse events. Correlation strength was computed using Spearman correlation analysis. * p-value < 0.05
(C) Correlation matrix for the co-occurrence of depicted cutaneous adverse events. Correlations are shown as relative co-occurrence (color-coded) and absolute counts of overlapping patients (size-coded).

(D) Kaplan-Meier plot depicting progression-free survival probabilities for patients who either developed poliosis only (excluding such with vitiligo co-occurrence), vitiligo (and co-occurrence of poliosis) or any other cutaneous adverse event during ICB. Median PFS is marked by black dotted lines and 95% CI intervals of the investigated groups are indicated by color-coded dotted lines. Numbers at risk provided at the bottom panel. Statistical significance was calculated using unadjusted log-rank test.
(E) Kaplan-Meier plot depicting progression-free survival probabilities for patients who either developed exclusively poliosis (excluding such with vitiligo co-occurrence), poliosis and vitiligo, vitiligo only or any other cutaneous adverse event during ICB. Median PFS is marked by black dotted lines and 95% CI intervals of the investigated groups are indicated by color-coded dotted lines. Numbers at risk provided at the bottom panel. Statistical significance was calculated using unadjusted log-rank test.
(F) Kaplan-Meier plot depicting overall survival probabilities for patients who either developed poliosis only (excluding such with vitiligo co-occurrence), vitiligo (and co-occurrence of poliosis) or any other cutaneous adverse event during ICB. Median OS is marked by black dotted lines and 95% CI intervals of the investigated groups are indicated by color-coded dotted lines. Numbers at risk provided at the bottom panel. Statistical significance was calculated using unadjusted log-rank test.
(G) Kaplan-Meier plot depicting overall survival probabilities for patients who either developed exclusively poliosis (excluding such with vitiligo co-occurrence), poliosis and vitiligo, vitiligo only or any other cutaneous adverse event during ICB. Median OS is marked by black dotted lines and 95% CI intervals of the investigated groups are indicated by color-coded dotted lines. Numbers at risk provided at the bottom panel. Statistical significance was calculated using unadjusted log-rank test.
(H) Forest plot summarizing results from multivariate Cox-regression analysis for PFS in patients with cutaneous adverse events including hazard ratios with 95% confidence intervals and results from statistical testing. Cutaneous adverse events were stratified into poliosis only, vitiligo including co-occurrence of poliosis and vitiligo and other cutaneous adverse events.
(I) Forest plot summarizing results from multivariate Cox-regression analysis for PFS in patients with cutaneous adverse events including hazard ratios with 95% confidence intervals and results from statistical testing. Cutaneous adverse events were stratified into poliosis only, vitiligo and poliosis co-occurrence, vitiligo only and other cutaneous adverse events.
*Abbreviations: * p-value < 0.05, ** p-value<0.01.*

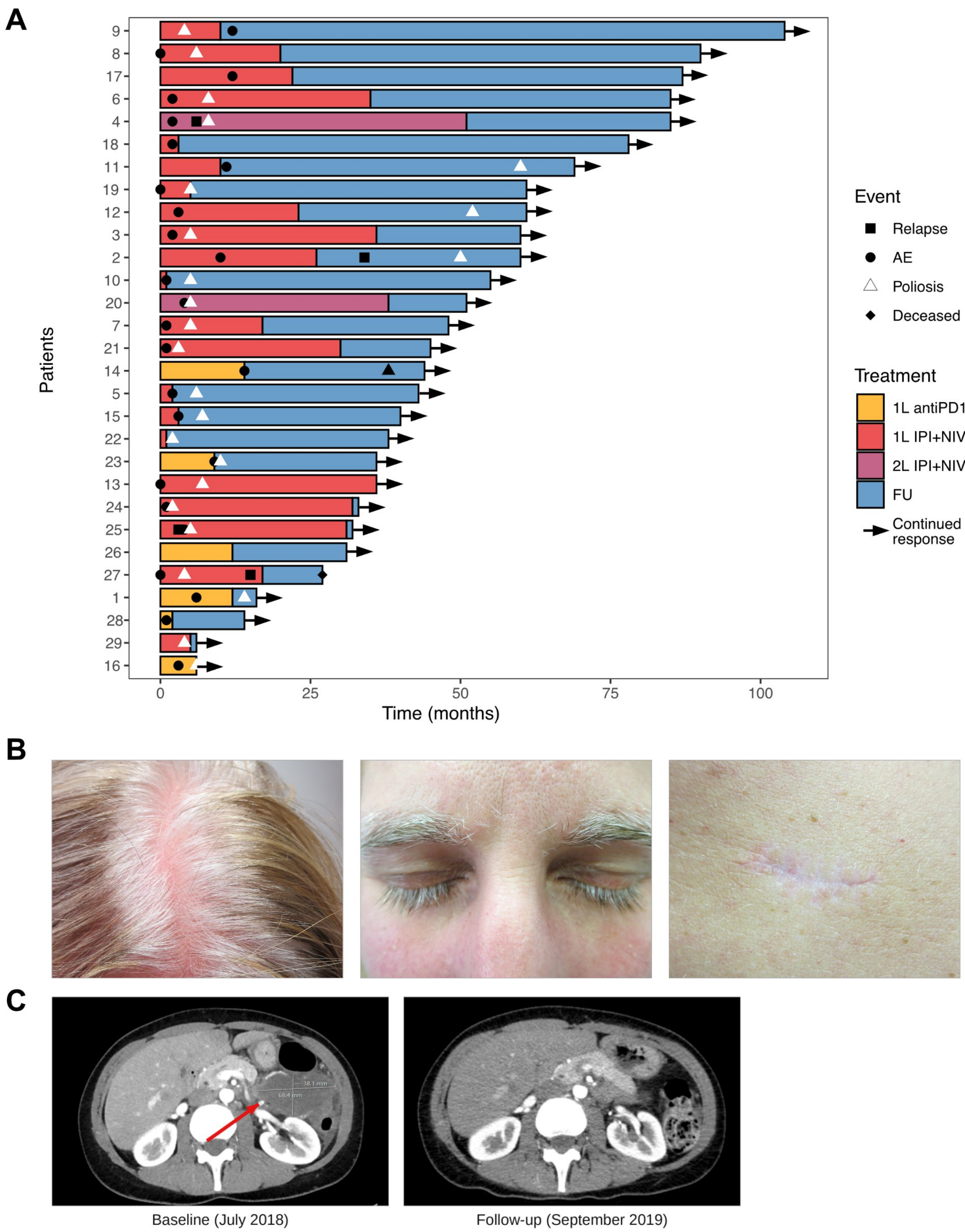

Supplemental Figure 4: Clinical presentation of poliosis following complete response to combined immunotherapy.

(A) Swimmer plot summarizing the individuals´ patient course over the time of treatment.
(B) A 51-year-old female patient showing poliosis of the hair (left), eyelids (center), and vitiligo of the scars (right) following combined immunotherapy.
(C) CT scans prior to ICB initiation and at follow-up reveal complete regression of gastrointestinal metastases upon ICB therapy.

Table S1. Univariable Cox-regression analysis for progression-free survival of the overall patient cohort (n=741).

Variables	Subgroups	HR	95% CI	*p*-value
Age (years)	>65 vs ≤65	0.99	0.83-1.19	0.953
Gender	Male vs Female	1.09	0.91-1.31	0.342
Ulceration	Yes vs no	1.09	0.88-1.35	0.414
BRAF status	wt vs BRAFV600	0.82	0.68-0.98	**0.032**
LDH elevated	Yes vs no	2.12	1.56-2.87	**<0.001**
Brain metastases	Yes vs no	1.23	0.01-1.51	**0.039**
Multifocal disease	Yes vs no	1.53	1.28 -1.83	**<0.001**
Treatment line	2nd vs 1st	1.47	1.19-1.83	**<0.001**
ICB agent	IPI vs anti-PD1 (ref)	1.61	1.02-2.54	**0.042**
	cICB vs anti-PD1	1.28	1.05-1.55	**0.012**
BOR (RECIST 1.1)	CR (ref)			
	PR vs	7.3	3.9-13.5	**<0.001**
	SD vs	15.0	8.2-27.4	**<0.001**
	PD	64.2	35.4-116.3	**<0.001**
irAE of CTCAE grade 2 or above	Cut.AE (ref)			
	No.cut.AE	1.8	1.4-2.2	**<0.001**
	No.AE.reported	2.13	1.7-2.7	**<0.001**
Cutaneous irAE stratification	Non.cut.AE (ref)			
	cut. irAE	0.7	0.5-0.8	**0.001**
	Vitiligo	0.5	0.3-0.7	**<0.001**
	Poliosis	0.12	0.05-0.3	**<0.001**

Table S2. Univariable Cox-regression analysis for overall survival of patients who developed any cutaneous adverse events (n=221).

**Variables**	**Subgroups**	**HR**	**95% CI**	***p*-value**
Age (years)	>65 vs ≤65	1.3	0.82-2.06	0.267
Gender	Male vs Female	1.08	0.66-1.75	0.762
Ulceration	Yes vs no	1.21	0.7-2.08	0.500
BRAF status	wt vs BRAFV600	1.33	0.81-2.20	0.265
Brain metastases	Yes vs no	0.7	0.4-1.24	0.223
Elevated LDH	Yes vs no	1.82	1.10-2.99	**0.019**
Multifocal disease	Yes vs no	1.40	0.86-2.28	0.175
ICB line	2nd vs 1st line	1.16	0.69-1.94	0.583
ICB agent	cICB vs antiPD1	0.97	0.6-1.57	0.908
BOR (RECIST 1.1)	PD (ref) vs rest	0.27	0.2-0.38	**<0.001**
Cutaneous AE	Other (ref)			
	Vitiligo	0.56	0.31-1.0	**0.050**
	Poliosis	0.06	0.01-0.46	**0.006**

Table S3. Baseline clinico-pathological features of patients who developed any cutaneous adverse events stratified by recruitment site.

Clinicopathological features	Discovery cohort	Validation cohort	*p*-value
Total number of patients	71	150	-
Median age at start of ICB in years (interquartile range, IQR)	64.0 (53-76.5)	65.5 (54-76)	0.690
Gender (%)			0.882
- male	44 (62.0)	95 (63.3)	
- female	27 (38.0)	55 (36.7)	
BRAF mutation status[1] (%)	33 (47.1)	52 (37.1)	0.181
LDH > 245 U/ml at baseline[2] (%)	31 (51.7)	76 (51.0)	0.10
Multifocal disease[3] (%)	24 (33.8)	90 (66.2)	**<0.001**
Melanoma brain metastasis (%)	24 (33.8)	28 (18.8)	0.181
Treatment-line of ICB (%)			0.069
- First	60 (84.5)	137 (91.3)	
- Second	11 (15.5)	13 (8.7)	
Immune-checkpoint blockade agents (%)			0.062
- IPI+NIVO	44 (62.0)	100 (66.7)	
- Anti-PD1	24 (33.8)	50 (33.3)	
- Ipilimumab	3 (4.2)	0	
Median Duration of ICB (95% CI)	7 months (2-21.5)	11 months (3.25-23.75)	**0.035**
Median follow-up upon start of ICB therapy (95% CI)	51 months (40-62)	51 months (39-59)	0.2
Median overall survival (95% CI)	NR	83 months (54-NR)	0.1
Deceased (%)	17 (23.9)	55 (36.7)	0.066

[1] BRAF mutation status was available in 206 patients
[2] LDH levels at baseline were available in 209 patients
[3] Data on multifocal disease at baseline were available in 207 patients
